# Are social interactions preferentially attended in real-world scenes? Evidence from change blindness

**DOI:** 10.1177/17470218231161044

**Published:** 2023-03-26

**Authors:** Mahsa Barzy, Rachel Morgan, Richard Cook, Katie LH Gray

**Affiliations:** 1 School of Psychology and Clinical Language Sciences, University of Reading, Reading, UK; 2 School of Mathematics and Statistics, University of Reading, Reading, UK; 3 Department of Psychological Sciences, Birkbeck, University of London, London, UK; 4 Department of Psychology, University of York, York, UK

**Keywords:** Social interaction perception, social perception, change detection, change blindness, inversion effect, real-world scenes

## Abstract

In change detection paradigms, changes to social or animate aspects of a scene are detected better and faster compared with non-social or inanimate aspects. While previous studies have focused on how changes to individual faces/bodies are detected, it is possible that individuals presented within a social interaction may be further prioritised, as the accurate interpretation of social interactions may convey a competitive advantage. Over three experiments, we explored change detection to complex real-world scenes, in which changes either occurred by the removal of (a) an individual on their own, (b) an individual who was interacting with others, or (c) an object. In Experiment 1 (*N* = 50), we measured change detection for non-interacting individuals versus objects. In Experiment 2 (*N* = 49), we measured change detection for interacting individuals versus objects. Finally, in Experiment 3 (*N* = 85), we measured change detection for non-interacting versus interacting individuals. We also ran an inverted version of each task to determine whether differences were driven by low-level visual features. In Experiments 1 and 2, we found that changes to non-interacting and interacting individuals were detected better and more quickly than changes to objects. We also found inversion effects for both non-interaction and interaction changes, whereby they were detected more quickly when upright compared with inverted. No such inversion effect was seen for objects. This suggests that the high-level, social content of the images was driving the faster change detection for social versus object targets. Finally, we found that changes to individuals in non-interactions were detected faster than those presented within an interaction. Our results replicate the social advantage often found in change detection paradigms. However, we find that changes to individuals presented within social interaction configurations do not appear to be more quickly and easily detected than those in non-interacting configurations.

## Introduction

Given the brain’s capacity limitations, incoming sensory information must be selected for further processing. Previous research has repeatedly shown that animate and socially relevant features of a scene, such as human faces/bodies and other animal species, are processed with a higher priority than non-social or inanimate features (Adolphs & Spezio, 2006; Bracco & Chiorri, 2009; Fletcher-Watson et al., 2008; New et al., 2007; Shiffrar et al., 2004). For example, evidence from visual search tasks show that human body/face targets are detected more quickly than other object targets (Doi & Ueda, 2007; Keys et al., 2021; Williams et al., 2005), and eye tracking experiments show that observers have an overall preference to fixate on social aspects over non-social aspects of a scene (Birmingham et al., 2008; Crouzet et al., 2010; Foulsham et al., 2010). These results suggest that our visual system is tuned to detect and process social information with a higher efficiency than non-social information.

Change detection paradigms (also known as Flicker paradigms) have been used to explore the allocation of visual attention. They show that under specific circumstances, our vision can fail to perceive salient changes that are easily noticed otherwise (Rensink, 2002). In this paradigm, original and altered versions of a scene are separated from each other using a brief blank screen, and are switched back and forth repeatedly, until the change is detected. Change detection under these circumstances can take some time, suggesting that both attention and awareness of the changed property are required for detection (Simons & Rensink, 2005). Hence, shorter response times to detect the change imply early allocation of attention to the changed area (Rensink et al., 1997). Using this paradigm, researchers have found that salient low-level features (Rensink, 2000; VanRullen, 2003) and salient objects (Henderson et al., 1999; Peterson & Berryhill, 2013; Shiffrar et al., 2004) are detected more quickly than other aspects of the display.

In change detection tasks, observers are quicker and better able to detect changes to social versus non-social stimuli (Bracco & Chiorri, 2009; New et al., 2007). New et al. (2007) used real-world, complex scenes to explore whether attention is directed differently to animate versus inanimate parts of a scene in a change detection task. They found that observers were quicker and better at detecting changes when they were made to animate objects, including both human and non-human animals, compared with inanimate objects, such as vehicles, buildings, and plants. To ensure that the results were not driven by differences in low-level visual features between the images in different conditions, New et al. (2007) also ran an inverted version of the task, where the images were shown upside down. Inversion has been used as a tool to control for low-level properties in face (Rossion, 2008; Valentine & Bruce, 1986), facial expression (Gray et al., 2013), and body (Bannerman et al., 2009) processing research, as well as in studies using complex scenes (Kelley et al., 2003; New et al., 2007). Inversion is used because low-level features are well-matched between inverted and upright versions of the stimuli, while the higher-level meaning is more difficult to extract when images are inverted than upright. In New et al.’s (2007) study, the between-category differences found in the upright version of the task were eliminated when participants completed the inverted version.

While previous research has focused on how individual faces and bodies are processed, there is growing interest in how observers process scenes containing multiple people (Bunce et al., 2021; Gray et al., 2017; Isik et al., 2017; Papeo et al., 2017; Quadflieg et al., 2015; Vestner et al., 2019). In visual search tasks, pairs of individuals arranged facing each other are detected faster than the same individuals arranged back-to-back (Papeo et al., 2019; Vestner et al., 2019, 2020; Vestner, Gray, & Cook, 2021; Vestner, Over, et al., 2021). It is thought that these facing stimuli are perceived as a social interaction (Papeo et al., 2019; Vestner et al., 2019). One view suggests that social interactions capture attention because of their importance in our navigation of the social world around us (Papeo, 2020). Similar conclusions have been drawn from the area of action perception. Evidence suggests that compared with non-interactive actions and/or actions from only one agent, meaningful interactive actions gain preferential access to awareness (Su et al., 2016), and are easier to discriminate when embedded in noise (Manera et al., 2011; Neri et al., 2006). Thus, these findings have led to the suggestion that people engaged in a social interaction are prioritised in the visual hierarchy over those that are not engaged in an interaction (Su et al., 2016).

A competing account is that social interactions only confer an advantage in attentional tasks because of the attentional cueing properties of the constituent faces and bodies (Vestner et al., 2020). This account suggests that interactants efficiently direct spatial attention using gaze, head, and body cues to a region of space between the interactants (Vestner et al., 2020). It suggests that if multiple cues are directing spatial attention to one location, it is not surprising that visual information near that region is processed quickly. Consistent with this account, pairs of arrows and desk fans—stimuli that are known to direct observers’ visuospatial attention when shown individually—are also found faster in visual search tasks when shown front-to-front rather than back-to-back (Vestner et al., 2020, 2022; Vestner, Over, et al., 2021).

In the studies exploring social interaction perception, social interactions have typically been defined as two bodies facing each other at an equal distance against neutral, sparse backgrounds. Considering that people are often found interacting with others, and the importance of social interactions in everyday life, it is important to explore whether social interactions are prioritised in more realistic scenes. A limited number of studies have investigated social interaction processing in real-world scenes (e.g., Birmingham et al., 2009; Skripkauskaite et al., 2022). Birmingham et al., 2009 recorded observers’ eye-movements while they viewed scenes including of one or three individuals; when three individuals were presented, they were either interacting or non-interacting. General scanning behaviour was not found to differ between the interacting versus non-interacting conditions; however, no data were provided on the time-course of the eye-movements, so it is not possible to tell if interactions were prioritised (i.e., fixated earlier) in relation to non-interactions. Skripkauskaite et al. (2022) published a study in which observers’ eye-movements were tracked while viewing real-world scenes of interacting or non-interacting dyads. They found that observers’ overt attention was more quickly drawn to dyads interacting than not interacting. However, there was no control for low-level stimulus factors in their experiment, making it difficult to discern whether the effects were driven by the interacting nature of the stimuli or low-level stimulus properties.

In the current experiments, we used a change detection task and real-world scenes to explore whether changes are detected faster when they occur to individuals versus objects, and the extent to which it is important if the individuals are presented within the context of a social interaction. To explore whether effects were driven by low-level stimulus properties, we included an inverted control condition. To confirm that changes to social aspects of scenes are detected faster than non-social aspects, in Experiment 1, participants were presented with scenes in which changes either occurred to a non-interacting individual or an object. The aim of this experiment was to see whether we could replicate previous findings (e.g., Bracco & Chiorri, 2009; New et al., 2007) using an online data collection method. We predicted that there would be faster and more accurate detection of changes for individuals than objects, but only when the images were presented upright. In Experiment 2, participants were presented with scenes in which changes either occurred to an interacting individual or an object. We aimed to see whether the prioritisation of social information is also present when individuals are specifically presented within a social interaction. Again, we predicted that there would be faster and more accurate detection of changes for interacting individuals than objects, but only when the images were presented upright. Finally, in Experiment 3, to directly compare the speed at which changes are detected for non-interacting and interacting individuals, participants were presented with scenes in which changes either occurred to a non-interacting or an interacting individual. If social interaction contexts are prioritised in complex visual scenes, we predicted that there would be faster and more accurate detection of changes to interacting individuals than non-interacting individuals.

## Methods

### Materials and design

The experiments used a 2 × 2 mixed design, with Target Type (Experiment 1: non-interacting individuals, objects; Experiment 2: interacting individuals, objects; Experiment 3: interacting individuals, non-interacting individuals) as a within-participant variable and Orientation (upright, inverted) as a between-participant variable. Participants were randomly assigned to either the upright or inverted version of the task, and all participants were presented with both types of targets in each experiment. The dependent measures were participants’ response times and accuracy scores to detect the changes.

**Figure 1.**
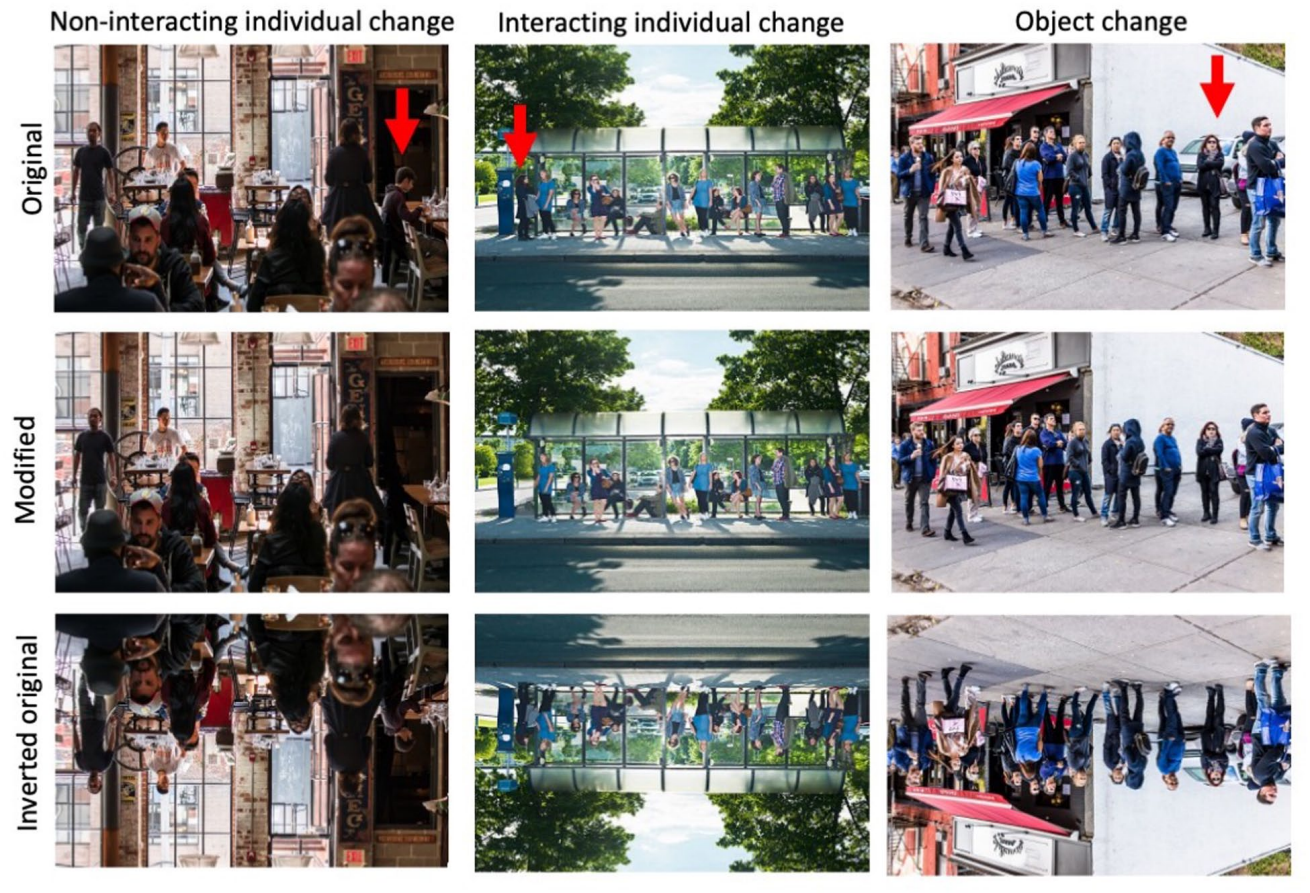
An example of a “non-interacting individual”, “interacting individual,” and an “object” change image, including the original image, and the modified, and inverted versions. Red arrows indicate the location of the changed individual/object.

The stimuli were natural social scenes, gathered from Google Images by searching phrases such as “people interacting in a park” or “people sitting in a restaurant.” The images were carefully considered to ensure that there were no copyright issues, image quality was good (minimum resolution = 738 × 466), none of the individuals pictured were looking at the camera, and finally there was a mix of people alone and interacting throughout the scenes. Seventy-two images were selected in total. There was no significant difference between the number of people depicted in the scenes across the three conditions, *F*(2, 69) = 1.60, *p* = .209, $\eta_{p}^{2}$ = .04. Using the photo editing software GIMP (GIMP 2.10.14, retrieved from http://gimp.org), a third of the images were edited to remove an object from the scene (e.g., bench, signpost), a third were edited to remove a lone person from the scene, and a third were edited to remove an individual who was engaging in a social interaction from the scene (24 images in each condition; see Figure 1 for examples). To confirm that the individuals in the scenes were recognised as interacting versus non-interacting, a sample of 21 raters indicated on a 7-point Likert-type scale the extent to which the target (i.e., the changed individual in the experimental trials) “was engaged in an interaction with another person.” Results from this rating study showed that targets in the “interacting individual” condition (*M* = 5.88, *SD* = 0.66) were perceived to be more highly engaged in a social interaction than those selected in the “non-interacting individual” condition (*M* = 1.87, *SD* = 0.50), *F*(1, 20) = 661.50, *p* < .001, $\eta_{p}^{2}$ = .97.

All 72 images were cropped to the same aspect ratio and then were resized to 700 × 420 pixels using MATLAB R2020b. An inverted version of each scene was then created through picture plane inversion. We also prepared a version of each scene with a grid of nine regions superimposed, which was used by participants to identify the location of the change on each trial. There were five practice trials which were different to the experimental stimuli and nine catch trials in which no changes were made between the two stimuli that were flickered. The stimuli for the practice and catch trials were obtained in the same way as the experimental stimuli.

### Procedure

All the experiments described were conducted online, an approach that is increasingly common. Carefully designed online tests of cognitive and perceptual processing can yield high-quality data, indistinguishable from that collected in the lab (Crump et al., 2013; Germine et al., 2012; Woods et al., 2015). The experiments were conducted online using the Gorilla Experiment Builder, a cloud-based research platform that allows researchers to create and deploy experiments online and collect precise behavioural/response-time data (Anwyl-Irvine et al., 2019, 2020). Participants were instructed to use only desktop computers or laptops.

First, participants were randomly assigned to the upright or inverted version of the task, and then they either completed the non-interacting individuals versus object version (Experiment 1), the interacting individuals versus object version (Experiment 2), or interacting individuals versus non-interacting individuals version (Experiment 3) of the task. Participants used the built-in screen calibration feature in Gorilla, where they adjusted the size of a rectangle to match the size of a credit card and gave their distance to the screen. Stimuli were presented at approximately 16 × 9.5° of visual angle. After providing informed consent, the study began with five practice trials (two non-interacting individual trials, two object trials, and one catch trial in Experiment 1; two interacting individual trials, two object trials, and one catch trial in Experiment 2; and two non-interacting individual trials, two interacting individual trials, and one catch trial in Experiment 3). Practice trials were followed by three blocks of 19 trials, each block including 3 catch trials and 16 experimental trials. After each block was an opportunity for a break. Each trial consisted of the original image presented for 300 ms, followed by a blank screen for 100 ms, followed by the edited image for 300 ms. These parameters were chosen as they are similar to those used in a previous change blindness study (Bracco & Chiorri, 2009). This sequence continued for up to 30 s, or until the participant pressed space bar to indicate they had identified a change. If they could not detect a change, participants were told to let the images time-out. At the end of each trial, participants were presented with the nine-grid image and asked to type the area in which the change occurred (1–9) or to type 0 if they detected no change. Participants were asked to respond as quickly and as accurately as possible. Accuracy was defined as the percentage of experimental trials in which participants correctly identified the location of the change. Response times were also recorded from picture onset to spacebar press, and only response times of correct responses were included in the analyses. All post hoc follow-up analyses described below were Bonferroni-adjusted. All raw data can be accessed at: https://osf.io/d83sz/?view_only=194e8f8506284813b19f86d46ddcdd72.

An a priori power analysis determined that a minimum of 19 participants would be needed to detect an effect size similar to that seen for the animate versus inanimate comparison (*d* = .68) found in New et al.’s (2007) study (with α = .05 and power of 95%; calculated using G*POWER; Erdfelder et al., 1996). We aimed for a sample size of at least 20 participants in the upright and inverted conditions for Experiments 1 and 2. We expected a smaller effect size in Experiment 3, as we were comparing between the two socially relevant conditions. Thus, we aimed to recruit at least 40 participants in the upright and inverted conditions, which would enable us to detect a minimum effect size of *d* = .58 with 95% power. As some participants were replaced (see details below), we liberally added participation slots to exceed our minimum sample size requirements. Participants across all experiments had normal or corrected-to-normal vision and gave informed consent. Ethical clearance was granted by the local ethics committee.

## Results

**Figure 2.**
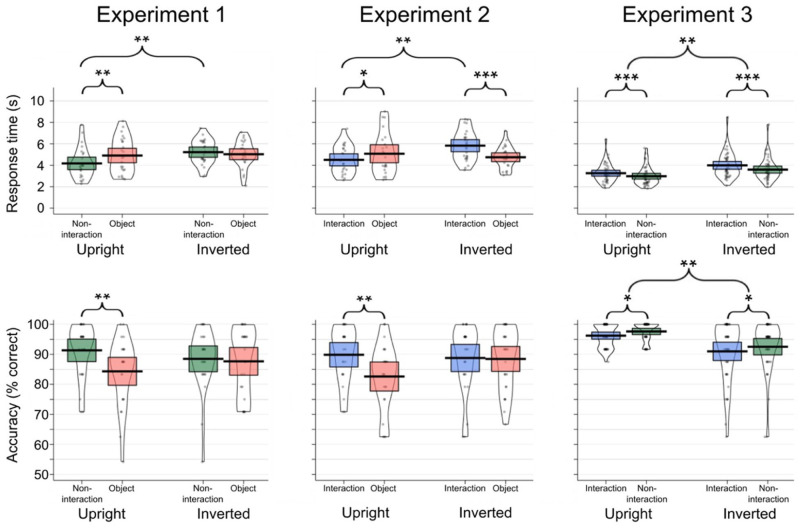
Change detection response times (top) and accuracy scores (bottom) for each condition for Experiment 1 (left panel), Experiment 2 (middle panel), and Experiment 3 (right panel). In each case, horizontal black lines denote the mean, and the rectangles denote the 95% confidence interval.

Response times and accuracy rates for each experiment are presented in Figure 2.

### Experiment 1

For Experiment 1, 50 participants (*M*_age_ = 21.12, *SD*_age_ = 5.59; 45 females, 5 males; 25 in the upright task, 25 in the inverted task) were recruited from the University of Reading in return for course credits. We ran 2 × 2 analyses of variance (ANOVAs) with Target Type (non-interacting individual, object) as a within-participant variable and Orientation (upright, inverted) as a between-participant variable on response times and accuracy. Seven participants were replaced having scored < 70% on the catch trials (*n* = 1) or having responded at chance levels (< 12/24 correct responses in each condition of the experimental trials; *n* = 6).

#### Response times

We found no main effect of Target Type, *F*(1, 48) = 2.59, *p* = .114, $\eta_{p}^{2}$ = .05, nor Orientation, *F*(1, 48) = 2.78, *p* = .102, $\eta_{p}^{2}$ = .06. However, in line with our predictions, the interaction between Target Type and Orientation was significant, *F*(1, 48) = 7.63, *p* = .008, $\eta_{p}^{2}$ = .14. For upright images, we found faster detection of changes to non-interacting individuals (*M* = 4,176 ms, *SD* = 1,413 ms) than objects (*M* = 4,907 ms, *SD* = 1,634 ms), *t*(24) = 3.09, *p* = .003, *d* = .48. When the images were inverted, changes to non-interacting individuals (*M* = 5,222 ms, *SD* = 1,160 ms) and objects (*M* = 5,029 ms, *SD* = 1,235 ms) did not differ significantly (*p* = .419). To further investigate the interaction, we explored the effect of Orientation in each of the Target Types. Changes to non-interacting individuals were detected faster when upright than inverted, *t*(48) = 2.86, *p* = .006; *d* = .81, whereas upright and inverted object changes did not differ significantly (*p* *=* .766).

#### Accuracy

There was no main effect of Orientation, *F*(1, 48) = .01, *p* = .924, $\eta_{p}^{2}$ < .01, but a significant main effect of Target Type, *F*(1, 48) = 7.59, *p* = .008, $\eta_{p}^{2}$ = .14, was subsumed within a significant Target Type × Orientation interaction, *F*(1, 48) = 4.70, *p* = .035, $\eta_{p}^{2}$ = .09. For upright images, accuracy was higher for changes that involved non-interacting individuals (*M* = 91.33, *SD* = 9.07) than objects (*M* = 84.33, *SD* = 11.30), *t*(24) = 3.48, *p* = .001, *d* = .68. This was not the case for the inverted images, where accuracy was similar for changes in non-interacting individuals (*M* = 88.50, *SD* = 10.44) and objects (*M* = 87.67, *SD* = 11.19), *p* = .680. Changes to non-interacting individuals and objects did not significantly differ in the upright versus inverted images (*p*s < .300).

### Experiment 2

For Experiment 2, 49 new participants (*M*_age_ = 23.04, *SD*_age_ = 7.22; 42 females, 5 males, and 2 “other”; 23 in the upright task, 26 in the inverted task) were recruited from the University of Reading in return for course credits. We ran 2 × 2 ANOVAs with Target Type (interacting individual, object) as a within-participant variable and Orientation (upright, inverted) as a between-participant variable on accuracy and response times. Ten participants were replaced having scored < 70% on the catch trials (*n* = 1) or having responded at chance levels (less than 12/24 correct responses on the experimental trials; *n* = 9).

#### Response times

There was no main effect of Target Type, *F*(1, 47) = 1.94, *p* = .171, $\eta_{p}^{2}$ = .04, nor Orientation, *F*(1, 48) = 1.81, *p* = .185, $\eta_{p}^{2}$ = 0.04. However, in line with predictions, the interaction between Target Type and Orientation was significant, *F*(1, 47) = 20.33, *p* < .001, $\eta_{p}^{2}$ = .30. In upright images, changes that involved interacting individuals (*M* = 4,504 ms, *SD* = 1,304 ms) were found faster than changes to objects (*M* = 5,074 ms, *SD* = 1,951 ms), *t*(22) = 2.14, *p* = .038, *d* = .34. For inverted images, however, changes involving interacting individuals (*M* = 5,827 ms, *SD* = 1,400 ms) were found significantly slower than changes to objects (*M* = 4,749 ms, *SD* = 1,017 ms), *t*(25) = 4.31, *p* < .001, *d* = .88. Changes were detected faster when interacting individuals were presented upright than inverted, *t*(47) = 3.41, *p* *=* .001, *d* = .98, but upright and inverted object changes did not significantly differ (*p* = .462).

#### Accuracy

There was no main effect of Orientation, *F*(1, 47) = .86, *p* = .359, $\eta_{p}^{2}$ = .02, and a significant main effect of Target Type, *F*(1, 47) = 5.73, *p* = .021, $\eta_{p}^{2}$ = .11, was subsumed within a significant Target Type by Orientation interaction, *F*(1, 47) = 4.80, *p* = .033, $\eta_{p}^{2}$ = .09. For upright images, accuracy was higher when responding to changes that involved interacting individuals (*M* = 89.86, *SD* = 9.39) than objects (*M* = 82.61, *SD* = 11.21), *t*(22) = 3.15, *p* = .003, *d* = .70. This was not the case for the inverted images, where accuracy was similar for changes in interacting individuals (*M* = 88.78, *SD* = 11.23) and objects (*M* = 88.46, *SD* = 10.29) (*p* = .883). Changes to interacting individuals and objects did not significantly differ in the upright versus inverted images (*p*s > .060).

### Experiment 3

For Experiment 3, 85 new participants (*M*_age_ = 28.74, *SD*_age_ = 11.08; 50 females, 35 males; 42 in the upright task, 43 in the inverted task) were recruited either from the University of Reading (*n* = 35; *M*_age_ = 21.14, *SD*_age_ = 5.26; 32 females, 3 males) or from Prolific (*n* = 50; *M*_age_ = 34.10, *SD*_age_ = 11.00; 23 females, 27 males; an online participant recruitment platform; www.prolific.co) to take part in return for course credits or financial compensation, respectively. Prolific was used to supplement the student sample in this study, as we had exhausted the local student participant pool. The number of Prolific participants in the upright (*n* = 24) and inverted (*n* = 26) conditions were well-matched. We ran 2 × 2 ANOVAs with Target Type (non-interaction individual, interaction individual) as a within-participant variable and Orientation (upright, inverted) as a between-participant variable on accuracy and response times. Four participants were replaced having scored < 70% on the catch trials.

#### Response times

There was a significant main effect of Target Type, *F*(1, 83) = 13.14, *p* < .001, $\eta_{p}^{2}$ = .14, where changes to non-interacting individuals (*M* = 3,290 ms, *SD* = 1,015 ms) were detected faster than changes to interacting individuals (*M* = 3,629 ms, *SD* = 1,105 ms). The main effect of Orientation was also significant, *F*(1, 83) = 11.56, *p* = .001, $\eta_{p}^{2}$ = .12, where changes were detected faster when the scenes were presented upright (*M* = 3,118 ms, *SD* = 766 ms) than inverted (*M* = 3,792 ms, *SD* = 1,038 ms). The interaction between Target Type and Orientation was not significant, *F*(1, 83) = 0.37, *p* = .546, $\eta_{p}^{2}$ < .01.

#### Accuracy

There was a significant main effect of Target Type, *F*(1, 83) = 6.04, *p* = .016, $\eta_{p}^{2}$ = .07, where changes involving non-interacting individuals (*M* = 95.05, *SD* = 7.17) were found more accurately than changes involving interacting individuals (*M* = 93.58, *SD* = 7.97). The main effect of Orientation was also significant, *F*(1, 83) = 12.94, *p* = .001, $\eta_{p}^{2}$ = .14, where accuracy was higher when responding to changes in upright (*M* = 96.93, *SD* = 3.06) than inverted (*M* = 91.76, *SD* = 8.79) scenes. The interaction between Target Type and Orientation was not significant, *F*(1, 83) = .02, *p* = .892, $\eta_{p}^{2}$ < .01.

## Discussion

Change detection paradigms are thought to capture the role of selective attention in identifying changes in visual displays. Using this paradigm, we conducted three experiments to investigate the speed of change detection when changes were applied to social versus non-social aspects of a scene. Using real-world scenes, participants had to find changes that occurred either by the removal of (a) an individual who was not engaged in a social interaction, (b) an individual who was interacting with another person/people, or (c) an object. First, we attempted to replicate previous findings by investigating whether changes to individuals were more quickly and accurately recognised than changes to objects. We next investigated whether the change detection advantage that has been reported for faces and isolated bodies compared with objects, could also be replicated for individuals in social interactions. Finally, we investigated whether changes to people in social interaction configurations were detected more easily than those in non-interacting configurations. An inverted version of the task was also included to discover whether any differences between conditions could be explained by low-level visual features.

The results of Experiment 1 showed that participants were significantly quicker and more accurate in finding changes to individuals versus objects in upright images. As we did not find a similar effect for inverted images, this indicates that the increased efficiency for detecting individuals compared with objects is driven by their high-level relevance, rather than image-specific differences, target differences, or low-level visual features. The results of Experiment 2 tell a similar story, where we found evidence that changes to individuals involved in interactions were detected quicker and more accurately than objects when presented in upright scenes. Again, results from the inverted condition make it clear that these effects are not driven by incidental differences between the images or targets. These two studies both replicate effects found in previous studies, where changes to socially relevant information are detected more quickly than other changes in complex scenes (Bracco & Chiorri, 2009; New et al., 2007). These findings also concur with previous effects that show attentional prioritisation of social stimuli using eye-tracking and visual search methods (Birmingham et al., 2008, 2009; Crouzet et al., 2010; Doi & Ueda, 2007; Keys et al., 2021; Williams et al., 2005).

In Experiment 2, participants were faster (*d* = .48) and more accurate (*d* = .68) in detecting changes to individuals in an interaction compared with objects when presented upright. The size of these effects were similar to the effects found in Experiment 1, where detection of non-interacting individuals was compared with objects (*d* = .34, and *d* = .70, for response times and accuracy, respectively). We directly compared the detection of non-interacting versus interacting individual changes in Experiment 3. Here, we did not find any change detection advantage for interacting compared with non-interacting individuals in the upright version of the task. This finding appears to contradict the suggestion that interactions are likely to be prioritised in the visual system (Papeo, 2020; Su et al., 2016).

With reference to the attentional hotspot account of social interaction processing (Vestner et al., 2020), our findings suggest that if attentional hotspots were elicited by the interactions in our stimuli, they were not strong enough to confer an advantage to change detection speed or accuracy for individuals presented within interactions versus individuals presented alone. Instead, we found an overall advantage for the detection of changes to non-interacting individuals, with participants being faster and more accurate when responding to individuals who were not engaged in social interactions than those who were. As the size of the effect was similar in both the upright and inverted versions of the task, the effect is unlikely to be driven by the high-level content of the images. It is more likely that this effect is driven by differences in low-level visual features of the images; for example, by differences between the images used in each condition, or differences in the targets that were selected. Overall, our results suggest that in complex, multi-agent scenes, a person presented within a social interaction context is not more salient than a lone individual.

The results indicate that change detection speed and accuracy to the socially relevant stimuli was disproportionately affected by inversion, whereas object changes were detected similarly in the upright and inverted versions of the task. The disproportionate effect of inversion to the recognition of faces and bodies versus objects has been extensively reported (Reed et al., 2003; Valentine & Bruce, 1986; Yin, 1969), and is thought to reflect the holistic processing of faces/bodies (McKone & Yovel, 2009; Searcy & Bartlett, 1996; Taubert et al., 2011). In terms of the detection of social stimuli, it has been theorised that we have an innate face detection mechanism, which not only draws us towards face-like configurations, but also helps us to detect other social cues such as eye contact and direct gaze (Johnson, 2005; Johnson et al., 1991). This mechanism is thought to be tuned to low-spatial frequency face-like configurations, and is thought to be orientation-specific, such that it cannot be engaged when the configurations are turned upside down (Gliga & Csibra, 2007; Johnson, 2005; Johnson et al., 1991).

While some researchers have used complex visual scenes to investigate social interaction processing (e.g., Birmingham et al., 2009; Skripkauskaite et al., 2022), many previous studies investigating how we process social interactions have used highly controlled images (e.g., where two identical bodies are posing at equal distance on neutral backgrounds; Bunce et al., 2021; Gray et al., 2017; Papeo et al., 2017, 2019; Vestner et al., 2019). The present study used real-world scenes which were not homogeneous. Complex visual scenes are most frequently seen in the real world, and we believe this is a strength of our paradigm. However, this necessarily gave us a lack of control over the image properties in each scene. By including an inverted version of the task, we could be sure that differences between the social relevance of the images were driving any effects, rather than low-level image properties or the changes that were made. A natural progression of this work would be to study the influence of individual differences, such as autistic traits and social anxiety, on the speed of detecting social changes. In general, the results of these experiments also suggest that change detection is a valuable paradigm with which to study social interaction perception.

We used online data collection for each of the experiments. We have found that online testing has produced clear, replicable results in visual search and attention cueing experiments (Vestner et al., 2022; Vestner, Grey, & Cook, 2021; Vestner, Over, et al., 2021), and studies of visual illusions (Bunce et al., 2021; Gray et al., 2020). However, this approach also has some well-known limitations. For example, it is not easy to control the testing environment, participants’ viewing distance, or their monitor settings. The results from Experiment 1 were consistent with well-known change-detection findings (Bracco & Chiorri, 2009; New et al., 2007) which gives us confidence that this did not affect our conclusions.

In conclusion, the current study demonstrated that, similarly to faces and individual bodies, changes to social interactions were also detected faster than changes to objects. A similar effect was also found for non-interacting individuals compared with objects, replicating the findings from previous studies. Furthermore, we found inversion effects for changes that were applied to non-interacting and interacting individuals, whereby they were detected more quickly when upright than inverted. No inversion effect was seen for objects, suggesting that the high-level, social content of the images was driving the improved change detection versus objects. When directly comparing changes for individuals in non-interactions versus interactions, we found that non-interacting individuals were detected slightly faster than those interacting. As this occurred across both upright and inverted versions of the task, it points to relatively low-level explanations. Overall, in a change-detection task utilising complex, real-world scenes, people presented within social interaction configurations were not found to be more salient than individuals presented alone.
